# Machine learning prediction of live birth after IVF using the morphological uterus sonographic assessment group features of adenomyosis

**DOI:** 10.1038/s41598-025-31013-1

**Published:** 2026-01-31

**Authors:** Sara Alson, Ola Björnsson, Emir Henic, Stefan R. Hansson, Povilas Sladkevicius

**Affiliations:** 1 Obstetric, Gynecological and Prenatal Ultrasound research, Department of Clinical Sciences, Malmö, Lund University, Malmö, Sweden; 2 Department of Obstetrics and Gynecology, Skåne University Hospital, Jan Waldenströms gata 47, S-205 02 Malmö, Sweden; 3 Reproductive Medicine Center, Skåne University Hospital, Malmö, Sweden; 4 Department of Energy Sciences, Faculty of Engineering, Lund University, Lund, Sweden; 5 Centre for Mathematical Sciences, Mathematical Statistics, Lund University, Lund, Sweden; 6 Department of Translational Medicine, Lund University, Malmö, Sweden; 7 Unit for Translational Obstetric Research, Department of Clinical Sciences, Lund University, Lund, Sweden

**Keywords:** Morphological uterus sonographic assessment (MUSA) group, XGBoost, Live birth, IVF/ICSI, Adenomyosis, Machine learning, Infertility, Medical research, Experimental models of disease

## Abstract

Predicting live birth after the first IVF/ICSI treatment is challenging, as many factors may interact to affect IVF/ICSI outcomes. Adenomyosis is one factor that impacts live birth rates. Machine learning algorithms have been shown valuable for detecting complex dependencies and predicting outcomes in different clinical settings. We aimed to develop a prediction model for live birth after IVF/ICSI treatment, using the Extreme Gradient Boosting (XGBoost) algoritm and incorporating the revised Morphological Uterus Sonographic Assessment (MUSA) group features of adenomyosis. We used a machine learning model based on data from 1037 women undergoing their first IVF/ICSI treatment between January 2019 and October 2022. The importance of each variable on the model was illustrated with the Shapley additive explanations algorithm (SHAP) variable importance. The prediction model was presented with the area under receiver operating characteristics curve (ROC). The proposed XGBoost model had a test AUC of 0.66 and accuracy of 0.59. S-AMH was the best variable for predicting live birth with a mean SHAP of 0.21, followed by a regular junctional zone as the best ultrasonographic variable, mean SHAP 0.13. The predictive ability of MUSA features in relation to live birth was limited. Additional variables should be included in future prediction models.

**Supplementary Information:**

The online version contains supplementary material available at 10.1038/s41598-025-31013-1.

## Introduction

In Vitro Fertilization (IVF) or Intra Cytoplasmic Sperm Injection (ICSI) treatments are widely used interventions for subfertility. However, assisted reproductive treatment (ART) does not guarantee success. Almost half of all couples that start ART will remain childless, even if they undergo multiple treatment cycles^1^. Predicting the chance of live birth (LB) after ART is desirable, to improve the individual counseling of couples. With adequate information, couples could weigh the chance of having a child against potential risks with the treatment and decide whether they are willing to accept the financial and emotional burden they might have to face.

However, predicting LB after the first IVF/ICSI treatment is challenging, as the outcome is influenced by several factors that need to be taken into account in clinically useful prediction models^2^. Among these factors is the presence of adenomyosis.

Adenomyosis is a hormone-dependent disease, characterized by ectopic endometrial tissue within the myometrium^3^. Adenomyosis has been linked to subfertility, and is believed to negatively impact ART outcomes, even if the precise mechanisms and causal links remain unknown^4^. A correct diagnosis is a prerequisite for studying the potential predictive value of adenomyosis on ART outcomes. Standardized diagnostic criteria to detect and describe adenomyosis on transvaginal ultrasonography (TVUS) have been suggested by the Morphological Uterus Sonographic Assessment (MUSA) group^5–7^. The revised MUSA criteria propose that features of adenomyosis should be divided into direct features, that are pathognomonic, and indirect, that are only suggestive of the disease^6^. Using these criteria, adenomyosis has been found to be present in approximately 20–30% of women undergoing ART^8,9^. However, the predictive value of the revised MUSA features on IVF/ICSI outcomes, particularly in relation to ovarian reserve parameters and the presence of endometriosis, has yet to be established.

Improved machine learning (ML) algorithms and increased computational power has led to an enhanced prognostic capability in different medical fields^10^. Prediction models based on supervised ML have by some been suggested to have superior predictive performance compared to conventional statistical methods^11–13^. Others have not found any superiority of ML over traditional methods in predicting reproductive outcomes^14^. A supervised ML algorithm called Extreme Gradient Boosting (XGBoost)^15^ is a powerful and widely used ML algorithm, known for its efficiency and effectiveness in predictive modelling tasks^15^. The XGBoost can analyze large datasets with numerous predictors and handle non-linear relationships between variables. This way, important features that contribute to the predictive performance of the model can be identified.

The aim of this pilot study was to explore the prognostic value of the revised MUSA features of adenomyosis^6^ for LB following the first IVF/ICSI treatment, using ML as a novel approach to capture potentially complex, non-linear associations.

## Materials and methods

### Study population and design

Between December 2018 and May 2021, we prospectively included 1037 women aged 25 - ≤39 years, who underwent their first IVF/ICSI treatment between January 2019 to October 2022 at the Reproductive Medical Center (RMC) at Skåne University Hospital, Malmö, Sweden. Eligible for publicly subsidized ART are women aged 25-≤39 years, non-smokers, with a Body Mass Index (BMI) (18-≤30 kg/m^2^), no common children with the present partner and with ≥ 1 year´s infertility. Women on current hormonal treatment were excluded, as this may alter the sonographic appearance of the myometrium.

Prior to starting ART, all women underwent a systematic two (2D) and three (3D) dimensional TVUS examination by the first author, using a Voluson 10 Expert (GE Medical systems, Zipf, Austria) high resolution ultrasound machine equipped with a 5–9 MHz transvaginal transducer (RIC5-9D). The ovarian Antral Follicle Count (AFC) was the sum of all follicles sized 2 to 10 mm. The uterus was measured in sagittal and transverse sections. The presence, extent (more or less than 50% of the myometrium), location (anterior, posterior, fundus or lateral) and myometrial layer (inner, middle or outer myometrium) of adenomyosis was assessed using the revised MUSA definitions^6^, as previously described^9^. Direct features of adenomyosis are subendometrial lines and buds, hyperechogenic islands and cysts in the myometrium, whereas indirect features are globular uterus, asymmetrical myometrial thickening, fan-shaped shadowing, translesional vascularity and an irregular or interrupted junctional zone (JZ)^6^. The presence of endometriosis or Pouch of Douglas obliteration was assessed using the International Deep Endometriosis Analysis (IDEA) group definitions^7^ and the sliding sign technique^16^, as previously described^8^.

The serum antimüllerian hormone (s-AMH) level (pmol/L) was analyzed. All women filled in a questionnaire regarding the presence or absence of typical symptoms suggestive of endometriosis or adenomyosis (dysmenorrhea, pelvic pain, dyspareunia, dyschezia, dysuria, hematochezia, or hematuria).

All women were treated according to either the Gonadotropin releasing hormone (GnRH) antagonist or agonist protocol, depending on patient characteristics or preferences, as previously described in detail^17^. The antagonist protocol is usually recommended as the first-line treatment, but the agonist protocol can be recommended for women assessed to be low responders or those with extensive adenomyosis or either large endometriomas or endometriosis-associated severe pain. Some women with severe endometriosis went through an ultralong downregulation with GnRH agonist (Enanton-Depot, Orion Pharma AB, Danderyd, Sweden or Synarela, Pfizer AB, Stockholm, Sweden) for 3–6 months prior to ART start, particularly when treatment start was delayed. Mature oocytes were either inseminated or injected with sperm, depending on semen quality. The Gardner blastocyst grading scale was used to assess embryos^18^ and ET was done either two, three (cleavage stage) or five days (blastocyst stage) after ovum pick up (OPU). Blastocyst stage transfer is preferred, but based on the growth rate, the size and cell-to-cell contact of the blastomeres, and degree of fragmentation, the embryologist can recommend cleavage stage transfer instead. Single ET is standard clinical practice. Any surplus good quality embryos (GQE): s, were cryopreserved on day 5–6. Frozen thawed ET (FET) was carried out in natural or hormone replacement cycles. All embryos from the index treatment cycle were used until LB, defined as the birth of a living child at > 22 gestational weeks, was achieved or no embryos remained.

### Statistical analyses

For statistical analyses, we used the statistical package IBM Corp. released in 2020. IBM SPSS Statistics for Windows, Version 29.0. Armonk, NY, USA.

### Model development

The modeling was performed using the XGBoost^15^. The variables included in the model are presented in Table 1. These were based on previous knowledge and evidence and included age, BMI, ovarian reserve parameters (s-AMH and AFC), presence of the revised MUSA features of adenomyosis, endometriosis as well as symptoms such as dysmenorrhea, dyspareunia, pelvic pain, dyschezia, dysuria, hematuria, and hematochezia.

**Table 1:** Variables included in the prediction model.

Variable
Age
s-AMH
BMI
AFC
Uterus height
Uterus lenght
Uterus width
Sliding sign
Endometrioma
Deep endometriosis
Dysmenorrhea
Dyschezia
Dyspareunia
Pelvic pain
Regular JZ
Irregular JZ
Interrupted JZ
JZ not assessable
Subendometrial lines and buds
Hyperechogenic islands
Myometrial cysts
Globular uterus
Fan-shaped shadowing
Translesional vascularity
Asymmetry
JZ not visible
Type^a^
Extent^b^
Myometrial layer^c^
Location^d^

AMH = antimüllerian hormone; BMI = Body Mass Index; AFC = antral follicle count; JZ = junctional zone.

^a^ Type = Focal or diffuse; ^b^ Extent = mild (≤ 25%), moderate (> 25-≤50%) or severe (> 50%); ^c^ Myometrial layer = Inner myometrium, middle myometrium, outer myometrium, or all myometrium; ^d^ Location = anterior wall, posterior wall, fundus, lateral wall.

To ensure that the model development was unbiased, and that the internal validation was independent, the data set was randomly divided into train- and test-set, with 80% of the data assigned for training and 20% assigned as the test set. The train and test split were performed in a stratified fashion, ensuring that both datasets had the same distribution of the outcome variable.

The training was performed using stratified 5-fold cross-validation, i.e., the training data was divided into 5 folds, where 4 folds were used for training the model, and the fifth was used for validation. This process was iterated until every fold had been used as the validation set. The hyperparameters of the XGBoost model were optimized using the open-source python library Optuna, allowing for automatic hyperparameter optimization^19^. Optuna was set to optimize the average of the area under the curve (AUC) from the validation sets. The XGBoost parameter “n_estimators” was set to a constant value of 500, and the models were trained using early stopping to limit overfitting.

An ensemble prediction approach was employed to predict the test set. This involves aggregating the predictions from five models created during cross-validation, specifically selecting the models that have the best performance during the cross-validation process. The final prediction for each patient in the test set was obtained by averaging the predictions generated by these selected models. Optuna was set to use Tree-structed Parzen Estimator (TPE), to sample the hyper-parameter space^20^. The initial hyper-parameter space is found in Table 2.

**Table 2:** The initial hyper-parameter space used for Building the model.

XGB Parameter	Range	Distribution
Max_depth	[1, 20]	Discrete uniform
Learning_rate	[0.001, 2]	Log uniform
Colsample_bytree	[0.05, 1]	Log uniform
Subsample	[0.05, 1]	Log uniform
alpha	[0.01, 10]	Log uniform
lambda	[1e-8, 4]	Log uniform
gamma	[1e-8, 4]	Log uniform
Min_child_weight	[0.2, 10]	Log uniform

XGB = Extreme Gradient Boosting¸ Max = Maximum, Min = minimum.

The table describes the XGboost parameters that were considered for tuning, their ranges along with the sampling distribution of the parameter space.

Given the size of the patient cohort in this study, there was a need to address unnecessary variables to reduce the dimensionality of the dataset and to simplify the model. To achieve this, we first removed binary variables with less than 20 observations in one of the categories. Deep endometriosis in different locations was classified as “deep endometriosis”. The second step was to find a suitable model and calculate the importance of the variables using Shapley additive explanation algorithm (SHAP)-values^21^. The SHAP is a method used for explaining individual predictions made by machine learning models^22^, providing a way to understand the contribution of each feature to the final prediction. Variables that were then shown to have a negligible contribution to the model were considered for removal. This was the dataset used for the final model.

The prediction models were evaluated, and results presented as the area under the receiver-operating characteristics (ROC) curve and accuracy of the evaluation and test sets. The evaluation metrics were calculated based on the predictions of all patients whenever they were used as evaluation set during the cross-validation. The threshold to dichotomize the predicted probabilities was decided using Youden’s J statistic based on the ROC curve for the evaluation set^22^.

Model performance was compared using the area under the ROC curve. Model interpretations were generated using SHAP by transforming model features into clinical variables and representing them as patient specific visualizations.

### Primary outcomes

The primary outcome was to explore the prognostic value of the revised MUSA features of adenomyosis on LB following the first IVF/ICSI treatment, using an XGBoost model. In addition, exploratory analyses were performed including embryo stage and FET to assess their relative contribution to LB prediction. As an exploratory post-hoc analysis, we also evaluated a bagged decision tree model to compare predictive performance with the primary XGBoost model. Details and results of these analyses are provided as Supplementary Material.

### Ethics statement

The study was approved by the Regional Ethical Review Board of Lund university, Lund, Sweden, on September 11, 2018, with a reference number 2018/555. The study was conducted in accordance with the tenets of the Declaration of Helsinki. Informed, written consent was obtained from all participants.

## Results

The background characteristics and IVF/ICSI results of the women included in the study are presented in Table 3.

**Table 3:** Background characteristics and IVF/ICSI treatment results for the 1037 women included in the study.

Parameter	Total cohort, *n* = 1037	Direct features of adenomyosis on TVUS		No direct features of adenomyosis on TVUS
		Not identified, *n* = 935	Identified, *n* = 102	Indirect features *n* = 188
Age, years	32.0 (4.0)	31.7 (3.9)	34.4 (3.8)	32.3 (4.1)
BMI, kg/m^2^	23.9 (3.4)	23.9 (3.4)	24.4 (3.2)	24.4 (3.5)
s-AMH, pmol/L	18 (19)	19 (20)	14 (14)	17 (19)
AFC, n	17 (11–26)	17 (11–26)	14 (7–20)	16 (8–23)
Endometriosis	231 (22.3)	190 (20.3)	41 (40.2)	124 (66.0)
Indication for IVF/ICSI				
Unexplained	462 (44.6)	411 (44.0)	51 (50.0)	82 (43.6)
Male	326 (31.4)	298 (31.9)	28 (27.5)	57 (30.3)
Mixed^a^	35 (3.4)	29 (3.1)	6 (5.9)	12 (6.4)
Tubal	79 (7.6)	72 (7.7)	7 (6.9)	19 (10.1)
Endometriosis	50 (4.8)	48 (5.1)	2 (2.0)	9 (4.8)
Oligo-/amenorrhea^b^	63 (6.1)	57 (6.1)	6 (5.9)	8 (4.3)
Other^c^	22 (2.1)	20 (2.1)	2 (2.0)	1 (0.5)
CLBR after first IVF/ICSI treatment	424 (40.9)	399 (42.7)	25 (24.5)	50 (26.6)

TVUS = Transvaginal ultrasonography; BMI = Body Mass Index; IVF = In Vitro fertilization treatment, ICSI = Intracytoplasmic Sperm Injection Treatment, CLBR = cumulative live birth rate.

Values are given as n (%) of each group of women, mean (SD) or median (interquartile range). ^a^ Mixed = male and female factors, ^b^Including women with polycystic ovarian syndrome, ^c^Other = single woman or same gender couples.

Direct features of adenomyosis were present in 102 (9.8%) women and indirect features without direct features were seen in 188 (18.1%) women. Endometriosis was detected in 231 (22.3%) women.

Women with direct features of adenomyosis required higher median (range) FSH-doses (2200 (1750–3300) IU compared to healthy women (1750 (1350–2550) IU), *p* < 0.001. They had fewer retrieved mature oocytes (mean (SD) 7.5 (5) versus 9 (7.0), *p* = 0.043) and less often transfer of blastocyst stage embryos (24/102, 34.3%) compared to women without (341/935, 49.2%, *p* = 0.009).

Women with direct features of adenomyosis had a lower LBR of 25/102 (24.5%) while women without any direct features had a LBR of 399/935 (42.7%), *p* < 0.001. Women with indirect features, without direct features, had a LBR of 50/188 (26.6%).

**Fig. 1:**
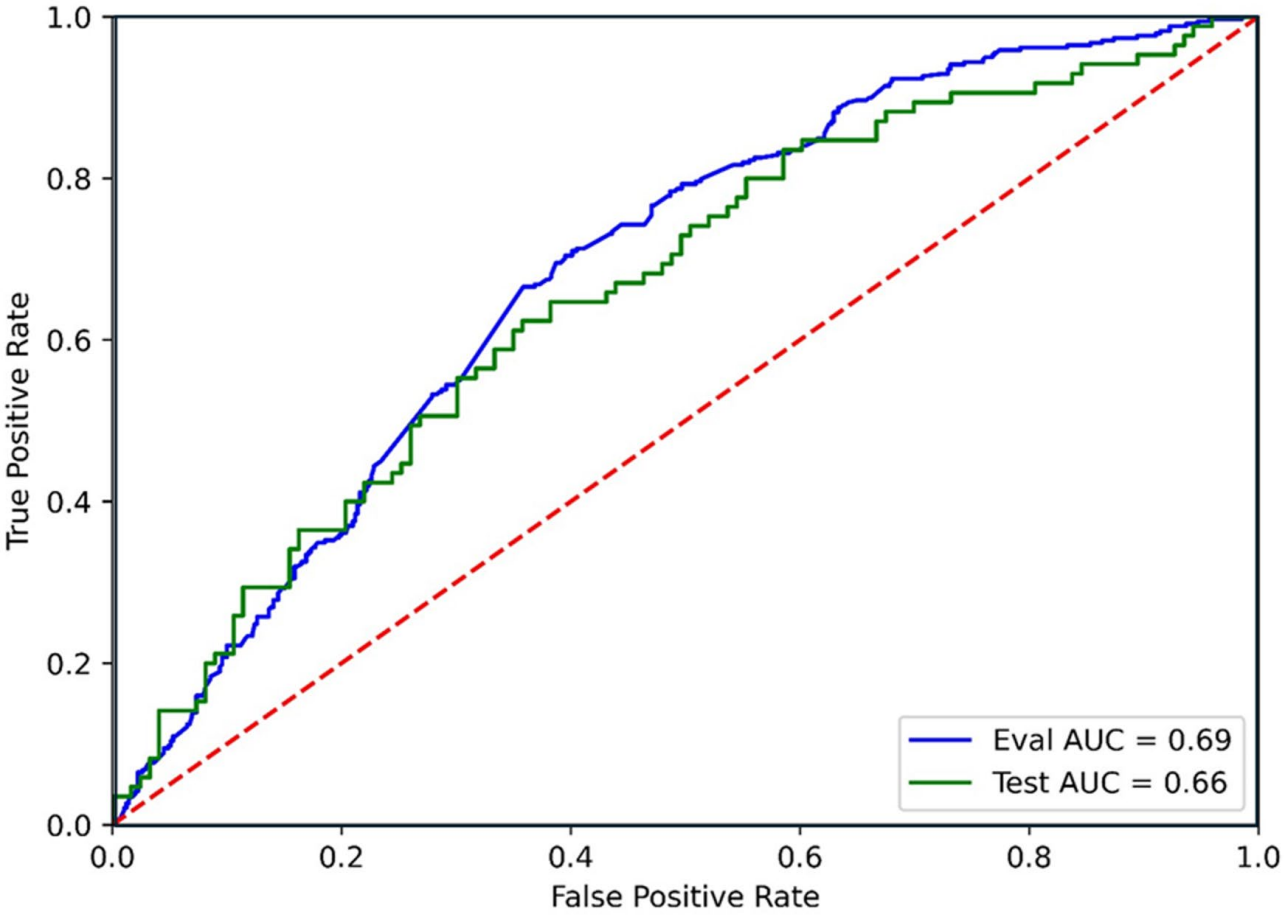
Receiver operating characteristics curves showing the area under the curve for prediction of live birth rate. LBR = Live birth rate; Eval = evaluation; AUC = area under the curve.

The best XGBoost model resulted in modest predictive performance, with a cross-validation accuracy of 0.64 and AUC of 0.69. The internal validation of the model on the test-set gave a lower accuracy of 0.59 and an AUC of 0.66, Fig. 1. F1-scores were around 0.55–0.57 and relatively higher recall (0.68) than precision (0.50) in the test set (Table 4).

**Table 4:** Threshold-dependent metrics for the model.

	Validation	Test
**F1-score**	0.553	0.574
**Precision**	0.555	0.496
**Recall**	0.55	0.682
**Accuracy**	0.64	0.59

**Fig. 2:**
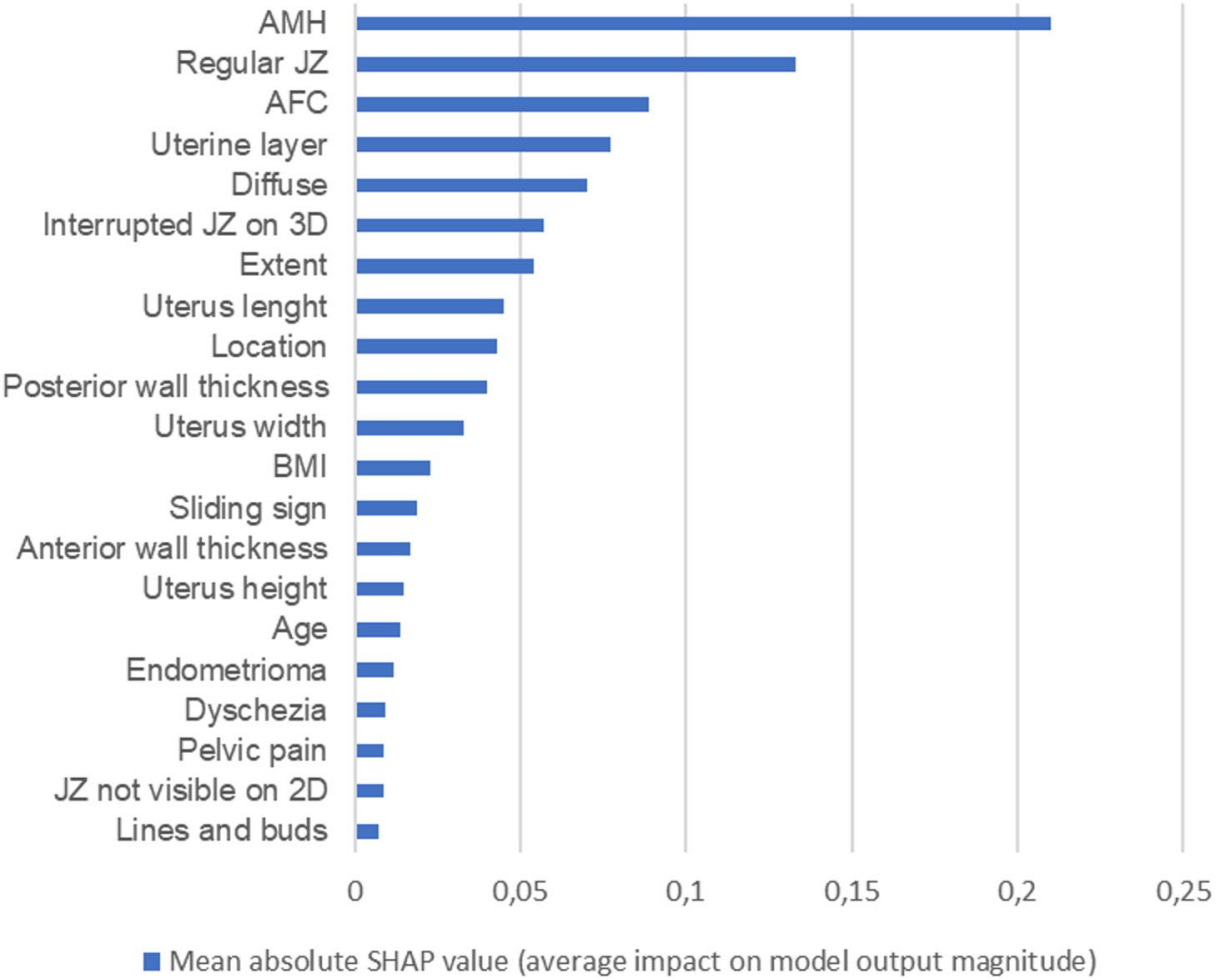
The importance of each variable on the prediction model for live birth, illustrated with the Shapley additive explanations algorithm (SHAP) variable importance. 3D = Three-dimensional; JZ = junctional zone; 2D = two-dimensional; BMI = Body Mass Index; AFC = antral follicle count; AMH = antimüllerian hormone; Extent = the extent (< or > 50%) of the revised Morphological Uterus Sonographic Assessment group fetaures of adenomyosis in the myometrium; pelvic pain = chronic pelvic pain. The most important variable has the highest mean of absolute SHAP values.

The importance of different clinical variables and direct or indirect MUSA features in the prediction model, ranked by the SHAP-values, is presented in Fig. 2. The variables with the best predictive ability in relation to LB were s-AMH (mean SHAP 0.21) and a regular JZ (mean SHAP 0.13). The best MUSA feature was an interrupted JZ, (mean SHAP 0.057), whereas the best direct MUSA feature was lines and buds (mean SHAP 0.007). The location of MUSA features in the myometrium had a mean SHAP-value of 0.077 for uterine layer. Variables with a negligible contribution to the model were dysmenorrhea, dyspareunia, translesional vascularity, unmeasurable JZ in 3D, myoma and deep endometriosis. The mean SHAP values for the different variables are presented in Table 5. The post-hoc model including embryo stage and FET shifted the feature ranking, with blastocyst stage (0.27) and cleavage stage (0.12) emerging as the dominant predictors, as presented in Supplemental Table S1. Adding these variables improved model performance, yielding an AUC of 0.75 compared with lower values in the pre-treatment model, Supplemental Figure S1.

**Table 5:** The importance of each variable on the model, illustrated with the SHAP values.

	Shap values
AMH	0.21
Regular JZ	0.133
AFC	0.089
Uterine layer	0.077
Diffuse	0.070
Interrupted JZ 3D	0.057
Extent	0.054
Uterus Length	0.045
Location	0.043
Posterior wall thickness	0.040
Uterus Width	0.033
BMI	0.023
Sliding sign	0.019
Anterior wall thickness	0.017
Uterus Height	0.015
Age	0.014
Endometrioma	0.012
Dyschezia	0.009
Pelvic pain	0.009
JZ Not visible 2D	0.009
Lines and buds 3D	0.007
Dysmenorrhea	0
Translesional vascularity	0
Myoma	0
Deep endometriosis	0

AMH = antimüllerian hormone; JZ = junctional zone; AFC = Antral follicle count; 3D = three-dimensional; BMI = Body Mass Index; 2D = two-dimensional, SHAP = the Shapley additive explanations algorithm.

As a post-hoc comparison, a bagged decision tree was also explored, yielding similar overall accuracy (65.9%) but lower sensitivity for LB compared with the XGBoost model. Given the limited added value and exploratory nature, detailed results are presented as Supplementary results.

## Discussion

In this pilot study, we used a ML model to find patterns that may not be recognizable by conventional regression methods. To the best of our knowledge, no previous study has incorporated the revised MUSA features in ML prediction models for LB after the first IVF/ICSI treatment.

The proposed XGBoost model showed a limited prognostic performance in predicting LB after the first IVF/ICSI treatment, with an internal validation accuracy of 0.59 and AUC of 0.66. S-AMH was the best variable with the highest SHAP value, whereas a regular JZ was the best ultrasonographic variable for predicting LB. Overall, direct MUSA features of adenomyosis, despite being pathognomonic for the disease, had a low predictive ability in relation to LB, which suggests that MUSA features alone are insufficient for clinically useful prediction of LB. Our suggested ML model had a similar performance as other ML models^14^, but was not superior to prediction models that were developed using conventional regression methods. Larger datasets with broader clinical variables will be required to improve prognostic utility.

Several attempts at developing prediction models for LB after IVF/ICSI treatment by using ML algorithms have been described^10,14,23^. In a recent study by Peng et al^14^, prognostic prediction models for the outcome LB after IVF/ICSI treatment were developed by using logistic regression and ML algorithms. In their study, the XGBoost model had a similar performance to our model, with an AUROC of 0.67.

The discriminative capacity of our model was modest, which is often the case for prediction models in reproductive medicine^24^. Recent evidence has shown no significant superiority of ML over traditional methods in predicting reproductive outcomes, and in some instances, statistical models have demonstrated greater ease of use and interpretability in clinical settings^14^. Various AI algorithms have been used in different studies to build prediction models for LB, which makes comparison between studies difficult. In one study that used four different models to predict LB after the first IVF, the XGBoost achieved the highest ROC AUC among all models, with a score of 0.73 on the validation dataset, which is similar to our results^23^. A potential limitation of the present study is that only one algorithm other than the XGBoost was tested. However, XGBoost was chosen to fit the size and characteristics of the specific dataset used in this study. Using models that are more suitable for other types of datasets would not necessarily have improved the performance.

When compared with conventional regression models, our ML model was not superior^25,26^. Previous logistic regression models using ovarian reserve parameters have achieved AUROC values around 0.68, which is similar to our findings^25^. Although AMH is only a modest predictor of LB when considered in isolation^27^, it remains one of the best baseline biomarkers of ART success, reflecting ovarian reserve and influencing the number of oocytes and embryos available for transfer or cryopreservation^28^. It is therefore likely that the contribution of the ovarian reserve in the model outweighs other female factors including adenomyosis, in determining the overall likelihood of LB after IVF/ICSI.

In our model, both AMH and AFC were important predictors, consistent with earlier studies showing ovarian reserve parameters as key determinants of LB^25, 26^. However, this contrasts with a retrospective study limited to single vitrified–warmed blastocyst transfers, where ovarian reserve markers were not predictive, likely reflecting differences between fresh and frozen cycles^29^. The number of embryos available for vitrification corresponds to the number of retrieved oocytes, which in turn reflects the ovarian reserve^27^.

Although age is often regarded as one of the strongest clinical predictors of ART success^30,31^, AMH emerged as the most influential feature in our SHAP analysis. This may partly reflect the study design, as women aged < 25 and ≥ 40 years were excluded due to Swedish IVF regulations, thereby reducing the variation and impact of age within the cohort. The relatively young age distribution of our cohort, with a median below 35 years, likely attenuated the predictive effect of age, as its adverse impact on IVF outcomes generally becomes evident only beyond this age. In contrast, AMH and AFC varied more widely and thus had stronger influence on the model. Importantly, AMH captures the quantity of oocytes but do not fully reflect their quality, and a woman with a preserved ovarian reserve at an advanced age is not necessarily equivalent in reproductive potential to a younger woman with a similar AMH level. Age encompasses oocyte competence and the risk of aneuploidy, which are central determinants of LB potential. It is also important to note that SHAP values describe feature contribution within the model and do not necessarily equate to absolute predictive value of the feature alone in clinical practice.

In our ML model, a regular JZ was the most important ultrasonographic variable for predicting LB. This aligns with existing theories that the JZ is vitally important for fertility and proper embryo implantation^32^. The JZ represents the subendometrial layer of the inner myometrium. A normal JZ is commonly defined as being regular in appearance on high-resolution ultrasound^33^, and is thought to reflect preserved myometrial architecture^34^. By contrast, a disrupted or thickened JZ on TVUS, as often seen in adenomyosis, has been associated with an abnormal inflammatory response within the myometrium, which in turn is strongly linked to local hyperestrogenism and progesterone resistance^35,36^. Associated dysfunctional uterine peristalsis and increased intrauterine pressure may affect sperm transport as well as embryo migration and implantation^37^, and lead to a defective spiral artery remodeling at decidualization^38^. In our ML model, the affected myometrial layer, regardless of whether the features of adenomyosis were direct or indirect, was more important than the actual presence of direct features. This aligns with the results of a previous study, in which women with features of adenomyosis located in the JZ had a more than threefold increased risk of miscarriage, whereas those with features of adenomyosis located in the outer myometrium, on the opposite, had a higher chance of ongoing pregnancy^39^. Features of adenomyosis present in the JZ may compromise successful embryo implantation and hence LB. Our results indicate that the presence of a regular JZ, which may be suggestive of absence of adenomyosis, in combination with a good ovarian reserve, could be reassuring for women who plan to undergo IVF treatment. However, they also suggest that reassessment of the current subdivision of MUSA features into direct and indirect features should be considered, as our findings indicate that this distinction does not necessarily translate into meaningful differences in prognostic performance.

A strength of this study is that the model was developed from multiple variables that were collected prospectively by an expert examiner, using well-defined disease criteria. Combining ultrasonographic findings of adenomyosis and endometriosis with subjective symptoms is another strength. The fact that the variables included in the model are available in most reproductive medicine centers around the world, and that most women undergo TVUS as well as an assessment of their ovarian reserve prior to starting ART, increases the generalizability.

Using the same experienced examiner ensures consistency in data collection and excludes any interobserver variability, which is considered a strength. However, this may limit the generalizability of the study, as limited interrater agreement in the ultrasonographic diagnosis of adenomyosis has been described^40^. This, however, may be more likely with inexperienced sonographers, and others have described good interobserver variability in the assessment of the JZ^41^.

Another strength is including fresh as well as frozen embryo transfers from the first IVF treatment cycle, which is in line with a widely accepted pre-treatment model^42^. We deliberately chose to include only parameters available prior to initiation of the first IVF/ICSI cycle, as our aim was to evaluate the pre-treatment prognostic potential of adenomyosis features, rather than to construct a fully comprehensive model incorporating treatment or laboratory variables. While this restriction may have contributed to the limited prognostic performance observed, it reflects the type of information available at the time of treatment decision-making. As expected, the inclusion of embryo stage and FET in a post-hoc analysis substantially increased predictive performance (AUC = 0.75) and dominated the feature ranking, consistent with their well-established role as key determinants of IVF outcome. Separating these groups could introduce bias given that the choice of ET protocol is closely linked to patient- and cycle-related factors such as ovarian reserve, age, presence of endometriosis and stimulation response. While these variables are not available at the pre-treatment stage, our exploratory analysis confirms their importance and underscores why our primary aim focused instead on pre-treatment prognostic markers.

A limitation of our study is the single-center design and the restricted age range of the cohort, which may limit generalizability. As age is both a major predictor of IVF outcome with substantial differences in success rates between women in their mid-20s and those in their late 30s^43^, and a risk factor for adenomyosis^44^, excluding older women could have influenced model performance. The fact that the prevalence of MUSA features themselves increases with age, makes age both a confounder and an important contextual factor. It is possible that excluding women ≥ 40 years may have influenced the predictive capacity of our ML model. Future studies should therefore model age more precisely, integrate additional predictors and recruit from multiple clinics with more diverse populations to assess whether the prognostic role of MUSA features varies across different age groups and clinical settings.

It is possible that different or less restricted feature selection could improve the model`s performance. Given the limited sample size, we opted for a pragmatic strategy combining sparsity filtering, clinically guided grouping, and model-based SHAP values. We included 25 variables in the dataset, and the selection of variables was based on subject knowledge and previous studies. A different variable selection may have rendered slightly different results^45^. In previous attempts at making prediction models for LB after IVF/ICSI, various predictive variables have been discussed^30, 46^. For example, male factors, such as semen quality parameters and sperm DNA Fragmentation Index, were not considered in this study, which may be considered a possible limitation. However, in several previous studies, male-related factors were found nonsignificant as predictors of LB^30, 47^, which was attributed to the fact that ICSI provides an effective treatment for male-related infertility. Future research with larger and more diverse datasets should explore systematic feature selection techniques (e.g., LASSO regularization or recursive feature elimination) to refine and possibly expand the set of predictors and potentially improve model performance and clinical applicability.

We may be criticized for including both AMH and AFC, as both reflect the ovarian reserve. The reason is that they capture slightly different biological aspects: AMH reflects granulosa cell function, whereas AFC represents a morphological count of recruitable follicles. Both are commonly used in clinical practice, and we therefore included them separately in our model.

Datasets predominantly composed of categorical and binary variables may present challenges for predictive modeling due to sparsity observed in the binary data. In this study, many variables had very few observations in one of the categories, which may have contributed to reduced model stability and performance. However, sparsity does not necessarily imply that variables are undesired and removing them solely due to sparsity may not be the most appropriate approach. Moreover, the XGBoost algorithm, which is relatively robust to sparse data structures through its ability to handle missing values and split-finding in sparse matrices, was chosen to mitigate this potential problem. Larger multicenter datasets with more balanced distributions across clinical features are needed in the future to overcome this challenge and validate our findings.

Overfitting is a common problem in ML, and particularly for small datasets this can lead to poor representation of the true underlying patterns in the data. However, the XGBoost method includes techniques to mitigate overfitting, and early stopping was employed when our model was developed.

Regarding the post hoc analysis, embryo developmental stage and transfer type (FET versus fresh) had the strongest influence on LB prediction, together with ovarian reserve parameters. These findings suggest that treatment-related and embryological factors outweigh pre-treatment uterine morphology in predicting outcomes.

While our model did not outperform conventional predictors such as age and ovarian reserve, it provides a formal evaluation of direct and indirect MUSA features of adenomyosis in the context of ART outcomes. Our findings highlight that, the ovarian reserve remains the dominant determinant of LB, and that both direct and indirect MUSA features of adenomyosis may contribute modest, prognostic information. Importantly, our results imply that further refinement of the revised MUSA criteria may be warranted. Our study should therefore be regarded as a proof-of-concept, demonstrating the feasibility of integrating MUSA features into prediction models. Larger studies, and evaluation of different predictors and ML algorithms^31^, will be required to determine whether the revised MUSA features of adenomyosis have true clinical utility in patient counseling or whether their value remains primarily theoretical.

Clinically, our results can be seen as reassuring: the presence of adenomyosis on ultrasound should not discourage couples from pursuing IVF/ICSI, as ovarian reserve remains the dominant determinant of success. This knowledge can inform patient counseling and individualized treatment planning, while ongoing research explores whether specific management strategies, such as higher-dose progesterone support or total-freeze protocols, may benefit women with adenomyosis.

In conclusion, when modeled with the XGBoost algorithm, the revised MUSA features of adenomyosis demonstrated limited prognostic value for LB after the first IVF/ICSI treatment in this pilot study. Although the presence of a regular JZ appeared reassuring, the overall predictive performance of MUSA features for LB was poor and comparable to existing approaches. Given that IVF outcomes are influenced by multiple interacting factors, future studies should include larger cohorts and a broader set of variables, and evaluate different ML approaches, to support the development of clinically useful prediction models. Moreover, reassessment of the current subdivision into direct and indirect MUSA features should be considered.

## Supplementary Information

Below is the link to the electronic supplementary material.

Supplementary Material 1

## Data Availability

The data underlying this article cannot be shared publicly due to ethical reasons and for the privacy of the participants *.* The data will be shared at reasonable requests to the corresponding author.

## Code availability

The code and algorithms used in the current study are available in the Zenodo repository, https://github.com/F-Ursus/ivf_lbr_prediction, ^48^.

The Syntax used in SPSS to generate the bagged decision tree is available as a Supplementary file, (Syntax used in SPSS to generate Decision tree).
