# Supplementary material for: Machine learning prediction of live birth after IVF using the morphological uterus sonographic assessment group features of adenomyosis
Source: Sci Rep. 2026 Jan 31;16:4324. doi: 10.1038/s41598-025-31013-1 (PMC12865173; doi:10.1038/s41598-025-31013-1)
Supplement: Supplementary file 1 — Supplementary Material 1 [file 41598_2025_31013_MOESM1_ESM.docx]

**Machine learning prediction of live birth after IVF using the Morphological Uterus Sonographic Assessment group features of adenomyosis**

Sara Alson, M.D.^a,b,c^*, Ola Björnsson^d,e^, Emir Henic, M.D., PhD.^c,f^, Stefan R. Hansson, M.D., PhD^b,g^, Povilas Sladkevicius, M.D., PhD.^a,b^

^a^Obstetric, Gynecological and Prenatal Ultrasound research, Department of Clinical Sciences, Malmö, Lund University, Sweden

^b^Department of Obstetrics and Gynecology, Skåne University Hospital, Malmö, Sweden

^c^Reproductive Medicine Center, Skåne University Hospital, Malmö, Sweden

^d^Department of Energy Sciences, Faculty of Engineering, Lund University, Lund, Sweden

^e^Centre for Mathematical Sciences, Mathematical Statistics, Lund University, Lund, Sweden

^f^Department of Translational Medicine, Lund University, Malmö, Sweden

^g^Unit for Translational Obstetric Research, Department of Clinical Sciences, Lund University, Sweden

***Corresponding author:**

Sara Alson

Department of Obstetrics and Gynecology, Skåne University Hospital

Jan Waldenströms gata 47

S-205 02, Malmö, Sweden

Phone: +46 739917318

E-mail: [sara.alson@med.lu.se](mailto:sara.alson@med.lu.se)

**Supplementary Figure 1.** Receiver operating characteristcs curves showing the area under the curve for prediction of live birth, including embryo stage and type of embryo transfer (fresh or frozen).


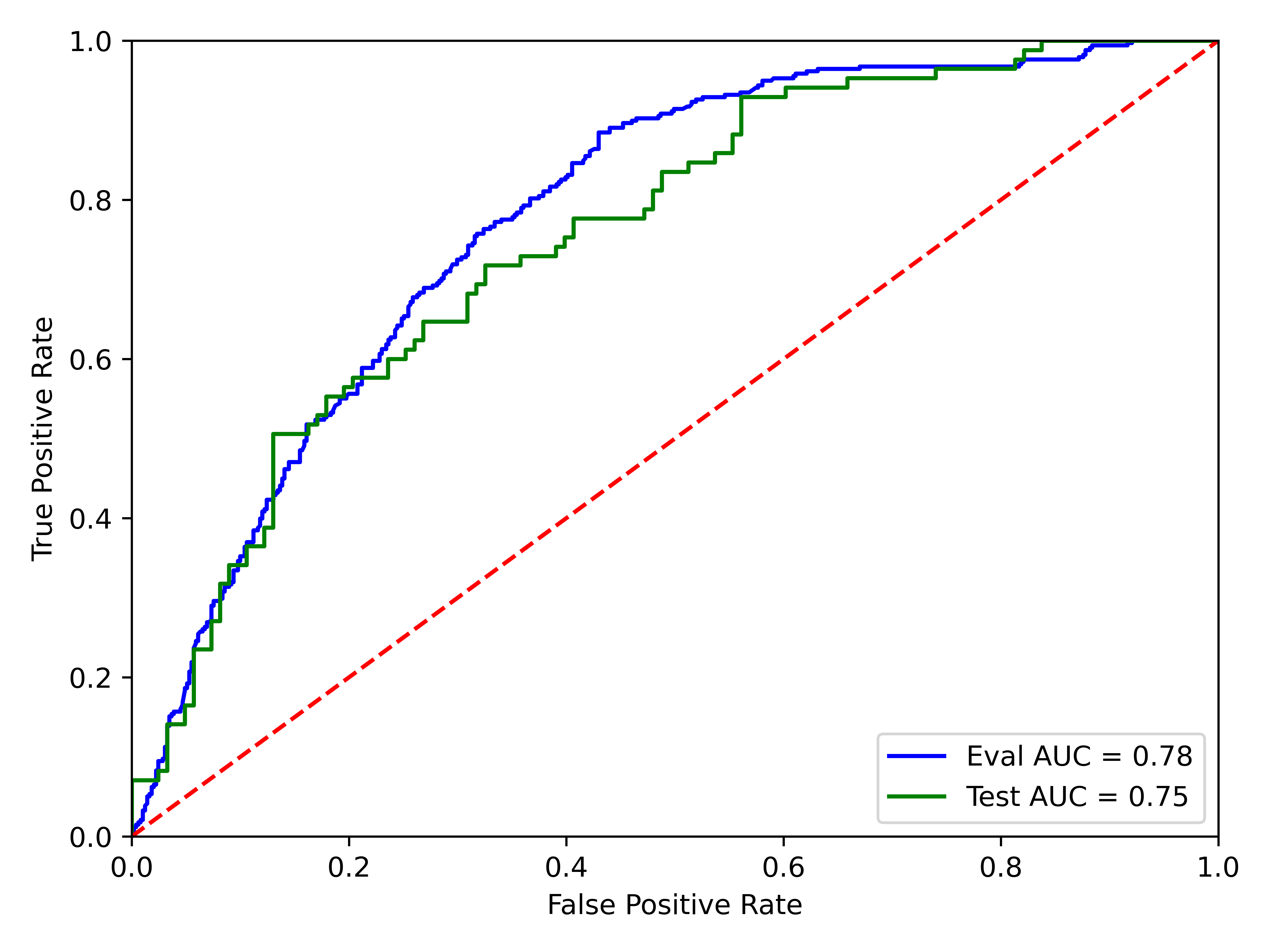


LBR= Live birth rate; Eval = evaluation; AUC= area under the curve.

**Supplementary Table S1.** The importance of each variable on the model, illustrated with the SHAP-values.

|  | **Shap values** |
| --- | --- |
| Blastocyst stage | 0.27 |
| Cleavage stage | 0.12 |
| AFC | 0.11 |
| AMH | 0.09 |
| FET | 0.06 |
| Diffuse | 0.05 |
| Interrupted JZ 3D | 0.034 |
| Location | 0.033 |
| Regular JZ | 0.032 |
| BMI | 0.030 |
| Posterior wall thickness | 0.025 |
| Uterus Height | 0.025 |
| Age | 0.021 |
| JZ Not visible 2D | 0.020 |
| Anterior wall thickness | 0.014 |
| Uterus Length | 0.011 |
| Uterine layer | 0.011 |
| Endometriosis | 0.009 |
| Uterus Width | 0.008 |
| Pelvic pain | 0.007 |
| Extent | 0.004 |
| Endometrioma | 0.002 |
| Dysmenorrhea | 0.002 |
| Dyspareunia | 0.002 |
| Translesional vascularity | 0.0008 |
| Myoma | 0.001 |
| Lines and buds 3D | 0.0005 |
| Dyschezia | 0 |
| Sliding sign | 0 |

AMH= antimüllerian hormone; JZ= junctional zone; AFC= Antral follicle count; 3D= three-dimensional; BMI= Body Mass Index; 2D= two-dimensional, SHAP= the Shapley additive explanations algorithm

**Supplementary Results: Bagged Decision Tree Analysis**

As a post-hoc exploratory analysis, we evaluated a bagged decision tree model. The first split was based on uterine layer, separating women with no adenomyosis features, inner myometrial involvement, and middle-to-outer myometrial involvement. Women with adenomyosis confined to the inner myometrium were least likely to achieve live birth (8.9%), compared with outer myometrium (24.5%) and absence of adenomyosis features (46.9%). Further splits were observed for AFC within the “no feature” group and posterior wall thickness within the “outer myometrium” group, with AFC 16–36 contributing to the largest proportion of correctly classified live births (33%). The overall accuracy of the model was 65.9%, with 77.5% of no-live-birth cases and 49.2% of live birth cases correctly classified (risk estimate 0.341, Standard Error 0.015). Given its comparable but less balanced performance relative to the main XGBoost model, the bagged decision tree results are presented here for completeness.

**Supplementary file, Syntax used in SPSS to generate Decision tree**

DATASET ACTIVATE DataSet2.

* Decision Tree.

TREE @1_Cumulative_LB [n] BY Age [s] BMI [s] AMH [s] DIE [n] Endometriosis [n] Dysmenorrhea [n]

Pelvic_pain [n] Dyspareunia [n] Focal_diffuse_tot [n] Uterine_layer [s] Extent [n]

JZ_3D_Interrupted [n] JZ_2D_Interrupted [n] Location_tot [n] JZ_2D_Regular [n] JZ_2D_Irregular [n]

Post_wall_thick [s] Ant_wall_thick [s] Uterus_Length [s] Uterus_Height [s] Uterus_Width [s]

Blastocyst_stage [n] Cleavage_stage [n] FET [s] Translesional_vascularity_2D [n] Myoma [n]

JZ_3D_Lines_and_buds [n] Myometrial_cysts_2D [n] Hyperechogenic_Islands_2D [n]

Subendometrial_lines_and_buds_2D [n] Dyschezia [n] Sliding_sign [n] AFC_Tot [s] Dysuria [n]

Hematochezia [n] Hematouria [n] Ear_sign [n] Enlarged_globular_uterus [n] Fanshaped_shadowing_2D

[n] JZ_3D_Cysts [n] JZ_3D_Hyperechogenic_dots [n] Endometrioma [n]

/TREE DISPLAY=TOPDOWN NODES=STATISTICS BRANCHSTATISTICS=YES NODEDEFS=YES SCALE=AUTO

/DEPCATEGORIES USEVALUES=[0 1]

/PRINT MODELSUMMARY CLASSIFICATION RISK

/METHOD TYPE=CHAID

/GROWTHLIMIT MAXDEPTH=AUTO MINPARENTSIZE=100 MINCHILDSIZE=50

/VALIDATION TYPE=NONE OUTPUT=BOTHSAMPLES

/CHAID ALPHASPLIT=0.05 ALPHAMERGE=0.05 SPLITMERGED=NO CHISQUARE=PEARSON CONVERGE=0.001

MAXITERATIONS=100 ADJUST=BONFERRONI INTERVALS=10

/COSTS EQUAL

/MISSING NOMINALMISSING=MISSING.
